# Chemotherapy-Induced Myopathy: The Dark Side of the Cachexia Sphere

**DOI:** 10.3390/cancers13143615

**Published:** 2021-07-19

**Authors:** Dean G. Campelj, Craig A. Goodman, Emma Rybalka

**Affiliations:** 1 Institute for Health and Sport, Victoria University, Melbourne, VIC 8001, Australia; dean.campelj@live.vu.edu.au; 2 Inherited and Acquired Myopathy Program, Australian Institute for Musculoskeletal Science (AIMSS), Victoria University, St Albans, VIC 3021, Australia; craig.goodman@unimelb.edu.au; 3 Centre for Muscle Research (CMR), Department of Physiology, The University of Melbourne, Parkville, VIC 3010, Australia; 4 Department of Medicine—Western Health, Melbourne Medical School, The University of Melbourne, Melbourne, VIC 3021, Australia

**Keywords:** cachexia, chemotherapy, exercise therapy, mitoprotection, muscle wasting, myopathy, pharmaceutical adjuvants, skeletal muscle

## Abstract

**Simple Summary:**

In addition to cancer-related factors, anti-cancer chemotherapy treatment can drive life-threatening body wasting in a syndrome known as cachexia. Emerging evidence has described the impact of several key chemotherapeutic agents on skeletal muscle in particular, and the mechanisms are gradually being unravelled. Despite this evidence, there remains very little research regarding therapeutic strategies to protect muscle during anti-cancer treatment and current global grand challenges focused on deciphering the cachexia conundrum fail to consider this aspect—chemotherapy-induced myopathy remains very much on the dark side of the cachexia sphere. This review explores the impact and mechanisms of, and current investigative strategies to protect against, chemotherapy-induced myopathy to illuminate this serious issue.

**Abstract:**

Cancer cachexia is a debilitating multi-factorial wasting syndrome characterised by severe skeletal muscle wasting and dysfunction (i.e., myopathy). In the oncology setting, cachexia arises from synergistic insults from both cancer–host interactions and chemotherapy-related toxicity. The majority of studies have surrounded the cancer–host interaction side of cancer cachexia, often overlooking the capability of chemotherapy to induce cachectic myopathy. Accumulating evidence in experimental models of cachexia suggests that some chemotherapeutic agents rapidly induce cachectic myopathy, although the underlying mechanisms responsible vary between agents. Importantly, we highlight the capacity of specific chemotherapeutic agents to induce cachectic myopathy, as not all chemotherapies have been evaluated for cachexia-inducing properties—alone or in clinically compatible regimens. Furthermore, we discuss the experimental evidence surrounding therapeutic strategies that have been evaluated in chemotherapy-induced cachexia models, with particular focus on exercise interventions and adjuvant therapeutic candidates targeted at the mitochondria.

## 1. Introduction

Chemotherapy constitutes a group of anti-neoplastic agents that were progressively discovered throughout the 19th century and became commonplace in oncological treatment as first-line or complementary therapeutic strategies for nearly all cancer types [1]. In general, chemotherapy targets cell cycle arrest through DNA-damage pathways that promote apoptotic cell death, although agents are heterogeneously stratified into different classes depending on their mode of action [2]. Independent of their different mechanisms, chemotherapies remain effective at inducing cancer cytotoxicity to abate the hyper-active neoplastic cell cycle [3]. Despite exerting anti-cancer efficacy, chemotherapy also elicits detrimental off-target side-effects to otherwise healthy cells due to their non-specific cytotoxicity [4]. Systemic toxicities are pervasive in the blood cell population as well as the central and peripheral nervous, gastrointestinal, cardiovascular, and integumentary systems [5,6,7,8]. Over the past decade, interest has developed concerning the non-specific impact of chemotherapy-induced toxicity on the skeletal muscular system [9,10,11]. Specifically, chemotherapeutic agents reduce body mass concurrent with skeletal muscle atrophy and dysfunction (referred to herein, as cachectic myopathy). These side-effects are clinically evident during patient deconditioning in the oncological setting [12], where weight loss and fatigue are two key debilitating events prominent in metabolic wasting syndrome, cachexia [13].

Cachexia is a multifactorial condition characterised by the loss of body mass and composition (highlighted by lean mass loss, with or without loss of fat mass) and progressive functional impairment [14]. The centrally afflicted organ in cachexia is skeletal muscle, which is driven by a multitude of factors including metabolic dysregulation; anorexia; systemic inflammation; and insulin resistance [15]. Skeletal muscle mass is an integral prognostic marker in cancer cachexia diagnosis—this is because increased adiposity de- sensitises the utility of body weight and body mass index (a crude indicator of body composition) during cachexia diagnosis [16,17]. Once instigated, cachectic myopathy propagates a vicious cycle involving increased risk of dose-related toxicities, which influences patient risk stratification and clinical decision making [18]. The result is compromised treatment efficacy (i.e., dose-reduction or treatment cessation) that increases the risk of morbidity and mortality [18]. Cachectic myopathy is the result of two synergistic insults from each of: (1) cancer–host interactions and (2) chemotherapy toxicity [15,19]. These effects can be acute, yet are most often life-long [20]. While the number of studies concerning cachexia is increasing exponentially, the proportion of these studies focusing on the impact of chemotherapy is far behind [9].

Overall, cancer cachexia represents a significant burden on patients and clinicians, with estimates suggesting that cachexia affects 50–80% of cancer patients and accounts for up to 20% of cancer deaths [21]. There are no current treatment options outside of standard nutrition interventions for body mass loss, which are largely unsuccessful [22,23]. Thus, cachexia is a significant unmet challenge in need of pre-clinical investigations to identify novel drug-targets and evaluate therapeutic interventions for clinical translation. Herein, we present a review of the literature surrounding experimental chemotherapy-induced cachexia, with particular focus on the skeletal muscle-specific side-effects and underlying mechanisms of myopathy. We also discuss the challenges associated with current experimental approaches used to investigate chemotherapy-induced cachexia and adjuvant strategies to protect against it.

## 2. Mechanisms of Chemotherapy-Induced Cachectic Myopathy

Chemotherapeutic agents work through different modes of action, which likely influences the degree to which they can induce cachectic myopathy. However, of the agents that do induce myopathy, there are some common underlying mechanisms [9]. It has been proposed that chemotherapy promotes systemic inflammation via the central nervous system, specifically through stimulation of the hypothalamus–pituitary–adrenal axis, to induce an adaptive illness response [24]. This response simultaneously induces the release of glucocorticoids and the production of pro-inflammatory cytokines (e.g., interleukin-1α and β, interleukin-6, and tumour necrosis factor-α (TNF-α)), which are both key events in the induction of skeletal muscle atrophy [25,26]. In particular, over-production of pro-inflammatory cytokines can directly induce skeletal muscle atrophy via engagement of membranous receptors and activation of a pro-catabolic transcription program [9,13]. Braun et al. proposes that instigation of the inflammatory milieu caused by chemotherapy increases activity of the gene, regulated in development and DNA damage response 1 (*REDD1*), which is associated with skeletal muscle atrophy [24]. REDD1 transcription regulates the adaptive stress response including the activation of stress-sensitive molecular targets, nuclear factor kappa-light-chain-enhancer of activated B cells (NF-κB), and mitogen activated protein kinase (MAPK) [27,28]. These two targets share common signalling pathways during oxidant-induced stress [29]. In particular, MAPK activity promotes phosphorylation of the NF-κB subunit, p65, resulting in NF-κB activation and the induction of skeletal muscle atrophy [29]. This atrophic response is achieved primarily through the ubiquitin-proteasome system (UPS)-mediated transcription of classic atrogenes, the E3 ubiquitin ligases, MuRF-1, and Atrogin-1 [30,31]. Additionally, it has been demonstrated by our laboratory and others that several chemotherapeutic agents can promote reactive oxygen species (ROS) production in C2C12 myotubes, which is associated with impaired myotube morphometry [32,33,34,35]. These data suggest that stress-sensitive molecular targets could be common signalling pathways in chemotherapy-induced cachectic myopathy. Furthermore, excessive ROS production is associated with the onset of mitochondrial dysfunction, an event postulated to be the crucial trigger for the induction of skeletal muscle wasting by chemotherapy [36].

As well as through directly targeting differentiated skeletal muscle tissue, chemotherapeutic agents also target myoprogenitor activity (i.e., satellite cell replication), which impacts skeletal muscle growth, repair, and turnover (as reviewed by us previously [37]). Many chemotherapeutic agents arrest cell cycling as their principal mechanisms of action against rampant cancer cell proliferation, resulting in visible side-effects in high-turnover cells including hair, bone marrow, skin, and gastrointestinal epithelium. It has recently emerged that muscle satellite (stem) cells, which normally undergo rapid proliferation and differentiation in response to muscle damage (e.g., inflammation) and growth factors (e.g., growth hormone and androgens) are also impacted, contributing to the net loss of muscle mass observed during cancer-related cachexia [38,39,40].

The research to date has predominantly focused on three key chemotherapeutic agents to study the mechanisms that govern chemotherapy-induced cachectic myopathy: the anthracycline, doxorubicin (DOX); the platinum-based alkylating agent, cisplatin (cis-diamminedichloroplatinum(II) (CDDP)); and the anti-metabolite, 5-fluorouracil (5FU). As such, we will further explore the current understanding of the connection between these stress-signalling cascades and the induction of cachectic myopathy in the context of specific chemotherapeutic agents and/or regimens.

### 2.1. Doxorubicin (DOX)

**Figure 1:**
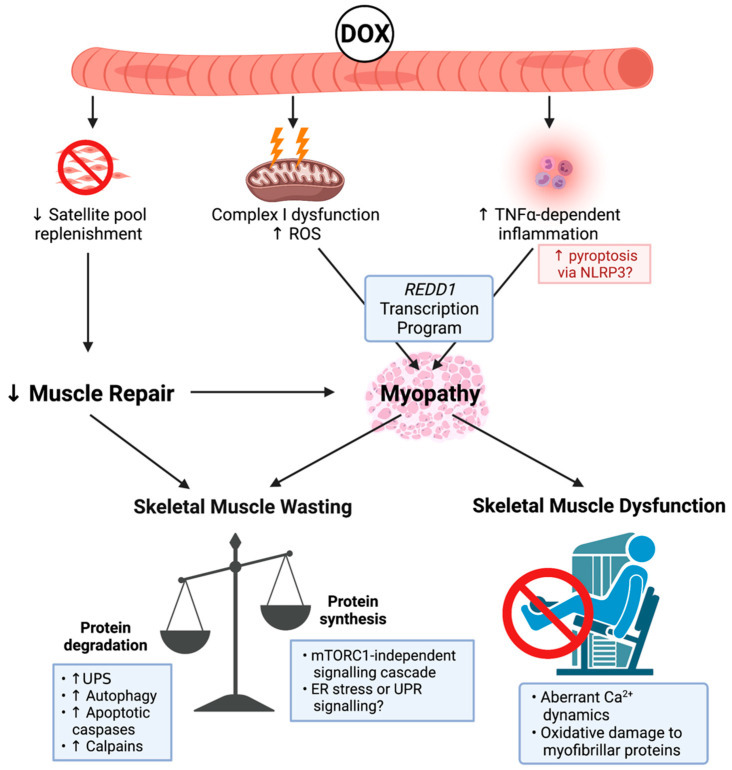
Known mechanisms of doxorubicin (DOX)-induced cachectic myopathy. DOX promotes reactive oxygen species (ROS) production primarily via Complex I dysfunction, which induces mitochondrial dysfunction and tumour-necrosis factor-α (TNF-α)-dependent inflammation, which can promote pyroptosis via increased nucleotide binding oligmerisation domain, leucine rich repeat-containing protein 3 (NLRP3) inflammasome formation, and activation of apoptotic caspases. This stimulates a regulation in the development and DNA damage response 1 (REDD1) transcription program, which overarches DOX-induced cachectic myopathy. DOX also reduces the replenishment of the satellite cell pool, which contributes to cachectic myopathy through impaired muscle repair. Underlying skeletal muscle wasting, DOX increases protein degradation via the ubiquitin-proteasomal system (UPS), autophagy, apoptotic caspases, and the calcium (Ca^2+^)-dependent proteases, calpains, while also reducing protein synthesis in a mammalian target of rapamycin complex 1 (mTORC1)-independent manner. While the exact mechanism has not been fully elucidated, endoplasmic reticulum (ER) stress or the unfolded protein response (UPR) signalling may be contributing factors. DOX also alters Ca^2+^ dynamics and promotes oxidative damage to myofibrillar proteins causing skeletal muscle dysfunction. Created with biorender.com (accessed on 6 July 2021).

DOX is a member of the anthracycline class of chemotherapies, and, as such, it elicits cytotoxicity via topoisomerase-II inhibition to induce DNA damage and cell cycle arrest [41]. Despite its potent anti-cancer efficacy, DOX is notorious for its cardiotoxic properties, which significantly limits its clinical utility [42]. Subsequently, there has been substantial interest in the effect of DOX on skeletal muscle health (summarised in Figure 1), particularly since skeletal muscle is an active compartment in the metabolism of DOX and its metabolites (e.g., DOX metabolite, doxorubicinol) is retained within skeletal muscle tissue for up to five days post-intravenous delivery [43,44]. The clinical implications of DOX administration are remarkable. Patients often present with debilitating fatigue, muscle weakness, and impaired ambulatory capacity [45,46]. At the cellular level, DOX treatment induces skeletal muscle dysfunction [47,48] characterised by reduced force production, impaired calcium (Ca^2+^) dynamics, and increased susceptibility to physiological fatigue [47,48,49]. Importantly, Gilliam et al. highlighted that these symptoms are dependent on the activity of pro-inflammatory cytokine, TNF-α [50]. This pro-inflammatory scenario has been shown to promote pyroptosis, a biological process of programmed cell death, which is characterised by the nucleotide binding oligmerisation domain (NOD), leucine rich repeat-containing proteins (NLR) family member, NRLP3 inflammasome formation, and activation of apoptotic caspases [51,52]. Furthermore, TNF-α mediates skeletal muscle contractile dysfunction through enhanced ROS production [53], although this is considered to be an additive effect to DOX-induced oxidative stress. DOX can directly stimulate ROS production through redox cycling at NADH dehydrogenase/Complex I of the mitochondrial electron transport chain [54]. As a result, ROS are produced, in particular superoxide anion radicals, via the reduction of DOX’s quinone moiety to an unstable semiquinone [54]. This redox cycling significantly elevates skeletal muscle hydrogen peroxide (H_2_O_2_) emission without perturbing homeostatic antioxidant buffering capacity [55,56,57] and alters bioenergetic efficiency by impinging on the functionality of respiratory complexes, leading to modifications that promote oxidative damage (e.g., lipid peroxidation) [56,58]. Recent findings from the Hulmi group suggest that DOX-induced skeletal muscle perturbations are predominately influenced by enhanced transcription of *REDD1* as part of the transcriptional program regulated by oxidative stress sensitive tumour suppressor protein, p53, a master regulator of cellular homeostasis [59,60,61]. Interestingly, there are few data connecting the promotion of inflammatory cytokines and oxidative stress from DOX to increased NF-κB activity in skeletal muscle, which is a downstream target of REDD1 transcription. Supriya et al. showed that DOX potentiated NF-κB activity in the skeletal muscle of diabetic mice [62]. The authors suggested that several mechanisms downstream of REDD1 were likely contributing [62].

Enhanced oxidative damage from DOX administration induces modifications that increase the catabolism of myofibrillar proteins, in particular actin and myosin, two integral proteins of the contractile apparatus [33,63]. The pro-catabolic signalling-cascade potentiated by DOX can activate multiple proteolytic systems including the UPS, autophagy, apoptotic caspases, and Ca^2+^-dependent proteases (i.e., calpains) [56,63,64]. The pro-catabolic shift in skeletal muscle protein balance has led to the consensus that DOX can cause skeletal muscle mass loss [11]. While DOX mechanisms are largely centred around proteolysis, there is also a rationale to suggest DOX impairs anabolism. This is highlighted by a reduced rate of protein synthesis independent of the classical signalling of mechanistic target of rapamycin complex 1 (mTORC1) [59], a key mediator of protein synthesis (for extensive review see [65]). There is also some evidence that DOX administration can impair the regenerative capacity of muscle through inhibiting satellite cell proliferation [66]. These data implicate alternative pathways that regulate protein synthesis during DOX administration. One such pathway was the activation of the endoplasmic reticulum stress/unfolded protein response signalling cascade, which can negatively regulate protein synthesis [67]. However, there were divergent responses between different types of striated muscle (i.e., heart, diaphragm, and limb skeletal muscles) and the expression of markers involved in this signalling cascade, elicited by DOX [55,68]. Thus, further research is required to enrich the understanding of the processes regulating the skeletal muscle protein balance during DOX treatment.

### 2.2. Cisplatin (CDDP)

**Figure 2:**
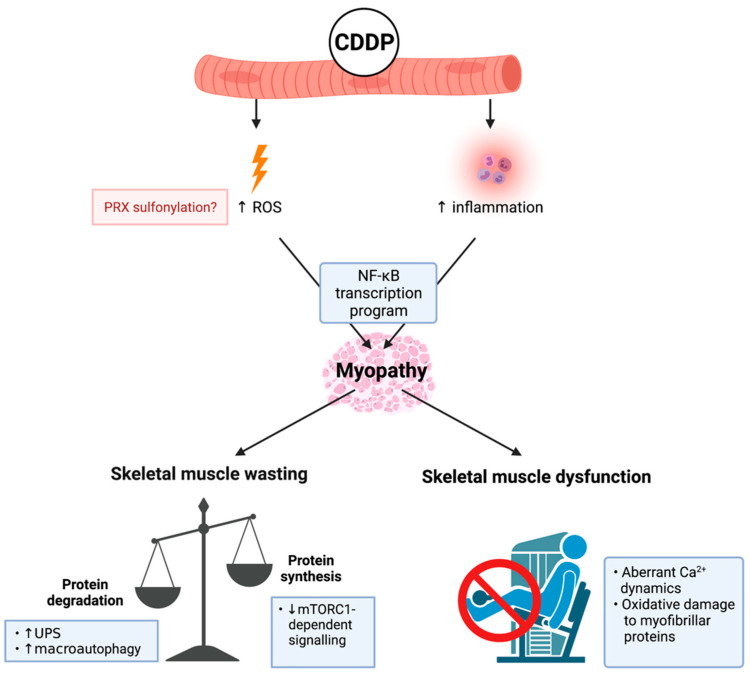
Known mechanisms of cisplatin (cis-diamminedichloropaltinum(II) (CDDP))-induced cachectic myopathy. CDDP promotes reactive oxygen species (ROS) production potentially through: (1) increased peroxiredoxin (PRX) sulphonylation; and (2) inflammation induced by pro-inflammatory cytokine mediated nuclear factor kappa-light-chain-enhancer of activated B cells (NF-κB) transcription program activation, which is a central mechanism of CDDP-induced cachectic myopathy. The underlying mechanism regulating CDDP-induced skeletal muscle wasting is increased protein degradation involving elevated ubiquitin-proteasomal system (UPS) activity and the promotion of macroautophagy. Additionally, there is evidence of reduced protein synthesis via mammalian target of rapamycin complex 1 (mTORC1)-dependent signalling cascades. CDDP induces skeletal muscle dysfunction through promoting aberrant calcium (Ca^2+^) dynamics and oxidative damage to myofibrillar proteins. Created with biorender.com (accessed on 6 July 2021).

CDDP is a platinum-based alkylating agent that enhances DNA damage via the aquation of its chloride ligands to form a highly reactive mono-aquated complex. This complex can bind with DNA residues to develop CDDP-DNA adducts, which induce DNA crosslinking and cell cycle arrest [69]. CDDP administration is associated with several toxicities, notably nephrotoxicity and neurotoxicity [70,71]. However, it also has deleterious impact on skeletal muscle (critically reviewed in detail previously [10,72]). CDDP was originally considered a potent inducer of negative protein balance—predominantly through impacting caloric intake (i.e., food consumption), which drives protein degradation to reduce body and skeletal muscle mass [73] (Figure 2). Importantly, Sakai et al. demonstrated that CDDP can drive protein degradation independently of caloric intake (through using pair-fed controls), in part, via an Akt and forkhead box O (FoxO)-dependent signalling cascade that enhances the transcription of atrogenes MuRF-1 and Atrogin-1 [74] while synergistically activating other constituents of the UPS [75]. CDDP also promotes the accumulation of autophagosomes (an indicator of autophagy-lysosome system dysregulation) via a similar, although currently undefined, Akt/FoxO3a-dependent mechanism [34,76,77]. Furthermore, CDDP suppresses protein synthesis via a protein kinase B (Akt)-dependent mechanism, which leads to the de-phosphorylation of p70^S6k1^, a downstream target of mTORC1 [78]. Thus, muscle anabolism signalling also appears to be impacted by CDDP (Figure 2). Damreuer et al. demonstrated that CDDP-mediated atrogene transcription occurs in response to trans-activation of NF-κB, specifically through the heterodimerisation of key subunit proteins, p50 and p65 [79]. Enhanced proteolysis induced by CDDP underscores skeletal muscle wasting, and this was initially thought to be the driver of functional decline [80]. However, recent findings from Conte et al. demonstrate that CDDP also dysregulates Ca^2+^ ion homeostasis, which is necessary for optimal skeletal muscle function [81]. CDDP increases the intracellular concentration of Ca^2+^, compromises Ca^2+^ dynamics, and de-sensitises the excitability of action potentials, resulting in reduced force production [82]. CDDP-induced oxidative stress is also thought to contribute to a dysfunctional contractile apparatus, although the underlying mechanisms are presently unclear. Sirago et al. proposed that CDDP drives H_2_O_2_ production and oxidative stress based upon evidence of increased peroxiredoxin (PRX) sulphonylation in skeletal muscle [77,83], which is also central to CDDP’s anti-cancer properties [84]. This mechanism has fascinating potential for therapeutic intervention given that PRX suppression is a key stimulant for NF-κB trans-activation [85], a critical target for CDDP-induced myopathy [9].

While CDDP is the predominate platinum-based chemotherapeutic agent experimentally evaluated for its effect on skeletal muscle health, our laboratory and others have demonstrated that analogues, carboplatin [86,87] and oxaliplatin (OXA) [88,89,90] can also contribute to the induction of cachectic myopathy. A complicating factor in the research surrounding platinum-based agents is the reliance on solvent, dimethyl sulphoxide (DMSO), to prepare experimental drug solutions. Using DMSO to deliver the chemotherapeutic agents CDDP and carboplatin suppresses their relative cytotoxicity in cell culture, whereas the cytotoxicity elicited by OXA is not impacted [91,92]. This highlights the complexity of interpreting the experimental data surrounding platinum-based complexes in comparison to vehicle control groups that do not disclose information regarding DMSO utilisation, an all too common feature in the accumulated literature thus far [92].

### 2.3. 5-Fluorouracil (5FU)

**Figure 3:**
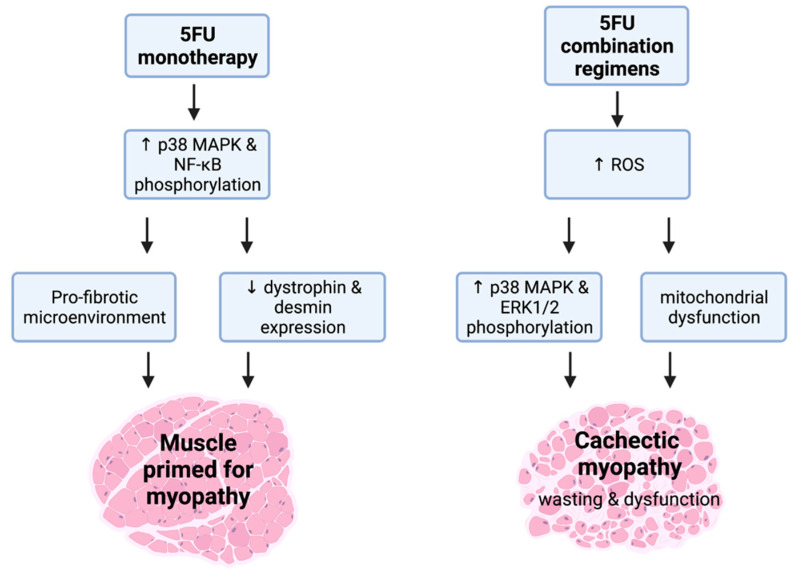
(5FU)-related cachectic myopathy. 5FU monotherapy promotes the phosphorylation of atrophic regulators, p38 mitogen activated protein kinase (MAPK), and nuclear factor kappa-light-chain-enhancer of activated B cells (NF-κB). These mechanistic targets are likely to be stimulated via signalling modulators including reactive oxygen species (ROS). 5FU does induce a pro-fibrotic skeletal muscle microenvironment and reduces the expression of the key cytoskeletal proteins, desmin and dystrophin, which suggests that 5FU primes muscle for cachectic myopathy. Interestingly, when additional chemotoxic insult to skeletal muscle occurs alongside 5FU such as in 5FU combination regimens, the induction of cachectic myopathy is observed. This is underscored by increased ROS production that stimulates the phosphorylation of p38 MAPK and ERK1/2 alongside mitochondrial dysfunction, leading to skeletal muscle wasting and dysfunction. Created with biorender.com (accessed on 6 July 2021).

5FU is a chemotherapeutic agent from the anti-metabolite class that elicits cytotoxicity via: (1) the misincorporation of nucleotides into RNA and DNA; and (2) the inhibition of the nucleotide enzyme, thymidylate synthase. This leads to both DNA and RNA damage and cell cycle arrest [93]. 5FU is primarily utilised against colorectal cancer as a backbone constituent of multi-agent regimens. However, it can elicit debilitating side-effects independently [93], emphasised by gastrointestinal toxicities such as mucositis and enteric neuropathy [94,95,96]. With respect to the side-effects of 5FU on skeletal muscle, there is mixed evidence as to whether it can independently drive the loss of mass and function [97,98,99]. Interestingly, VanderVeen et al. demonstrated that 5FU administration impairs the homeostatic coordination of skeletal muscle repair and remodelling through reducing M1-like macrophage abundance [98]. This suggests that 5FU dysregulates monocyte recruitment and drives a shift towards a pro-fibrotic skeletal muscle microenvironment [98] (Figure 3). The Bonetto group have extensively demonstrated that the 5FU-based combination regimen, FOLFIRI [5FU, leucovorin (LV), and irinotecan (IRI)], can drive cachectic myopathy, with several mechanisms explored [35,100,101,102,103]. In skeletal muscle, FOLFIRI: (1) promotes the phosphorylation of MAPK isoforms, p38 and ERK1/2; (2) increases serum ROS levels; (3) reduces mitochondrial number and size; and (4) downregulates the expression of protein markers indicative of mitochondrial maintenance and turnover (i.e., biogenesis, fission, and fusion) [35,101,102]. Despite FOLFIRI inducing cachectic myopathy, the 5FU-based combination regimen FOLFOX [5FU, LV, and OXA] has limited impact on skeletal muscle [35]. While these data may be accounted for by methodological specifics concerning the treatment timeline and/or dosages of the FOLFOX constituents, it is an interesting observation when considered in context of findings from our laboratory. We have demonstrated that IRI (constituent of the FOLFIRI regimen) monotherapy induces cachectic myopathy [104], which is characterised by reduced expression of dystrophin, a key structural protein that connects the sarcolemma to the actin cytoskeleton and maintains cytoskeletal integrity [104]. Similarly, we recently demonstrated that 5FU monotherapy also reduces dystrophin protein expression, in addition to desmin, an intermediate filament that provides stability to sarcomeres. However, these cytoskeletal protein changes are not associated with overt cachectic myopathy or loss of function [99]. These findings highlight that: (1) 5FU may prime skeletal muscle for myopathy by reducing the abundance of key cytoskeletal structural proteins; and (2) these events (i.e., loss of dystrophin and other cytoskeletal proteins) apparently precede alterations to skeletal muscle mass or function (Figure 3). Our work suggests that cytoskeletal proteins may have potential as early biomarkers for chemotherapy-induced cachectic myopathy, although this requires further investigation to be confirmed. 

### 2.4. Other Chemotherapeutic Agents

Several other chemotherapeutic agents have been investigated for their impact on skeletal muscle, albeit to a far lesser extent than DOX, CDDP, and 5FU. Distinct from platinum-based alkylating agents, chemotherapies arising from the alternative alkylating agent classes such as nitrosourea (i.e., cystemustine (CMN)) and nitrogen mustard (i.e., cyclophosphamide (CYP)) have also been investigated for their effect on skeletal muscle [73,105,106,107]. Interestingly, in cancer-free mice, CMN acutely reduces body mass, and, while post-chemotherapy catch-up growth is evident, skeletal muscle mass does not completely recover [106]. However, in tumour-bearing mice, the effect of CMN manifests differently. CMN treatment (10 days) reduces skeletal muscle mass, albeit, paradoxically, concurrent with enhanced protein synthesis and reduced proteasome-dependent proteolysis at the molecular level [105,106]. These data perhaps reflect an ongoing, yet unsuccessful, attempt to recover skeletal muscle mass in response to CMN treatment. Similar to CMN, CYP administration induces an acute loss of body mass followed by compensatory catch-up growth in cancer-free mice, but skeletal muscle mass loss is only mildly impacted [107]. However, CYP reduces ambulatory capacity and skeletal muscle adenosine triphosphate (ATP) production through impairing mitochondrial function [107]. CYP may also impact skeletal muscle through inducing neutropenia (CYP is used experimentally to generate rodent models of neutropenia) [108], possibly through impacting skeletal muscle repair and remodelling efficiency. A similar effect has been reported for 5FU [98].

Gemcitabine (GCB) is a chemotherapeutic agent from the anti-metabolite class, which has often been experimentally investigated as part of a combination regimen with CDDP consistent with its clinical utility for the treatment of metastatic cancers [109,110,111]. Given the extensive evidence supporting that CDDP treatment induces cachectic myopathy [10,72], it is currently unclear as to what extent (if at all) GCB contributes. Current evidence demonstrates that the CDDP and GCB combination potentiates tumour-induced skeletal muscle mass loss and proteolytic activity, while GCB alone has no impact [112,113,114]. This does not rule out the possibility that GCB exacerbates the effects of CDDP in skeletal muscle. Another chemotherapeutic agent from the anti-metabolite class is methotrexate (MTX), which is not only utilised in cancer, but in hyper-inflammatory conditions such as rheumatoid arthritis [115]. There are no data available surrounding the effect of MTX on skeletal muscle health in cancer-based models. However, in a mouse model of diabetes, MTX was shown to elicit benefits on skeletal muscle glucose metabolism [116,117]. These findings suggest that chemotherapeutic agents from the anti-metabolite class display a modest myotoxic profile compared to other chemotherapy classes.

Mitotic inhibitors are a class of chemotherapeutic agents that have been investigated for their effect on skeletal muscle, in particular taxanes and vinca alkaloids [118]. These drugs are known for their microtubule de-stabilising properties and potent neurotoxicity [119,120,121]. Docetaxel (DTX) is a taxane-based chemotherapy that demonstrably reduces skeletal muscle mass. This effect is evidenced by serum markers of malnutrition and inflammation, and a pro-catabolic transcriptional program, yet skeletal muscle contractile function is not affected [122,123]. Paclitaxel (PTX) is another taxane-based agent, albeit not extensively investigated with respect to skeletal muscle. Ramos et al. highlighted the capacity of PTX to alter skeletal muscle microtubule architecture observed through α-tubulin disorganisation [118]. Additionally, alterations to microtubules by PTX are suggested to be underpinned by impaired adenosine diphosphate (ADP)-dependent bioenergetics via the binding of tubulin to the mitochondrial ADP/ATP exchanger voltage-dependent anion channel (VDAC) [118]. Ramos et al. also studied the vinca alkaloid, vinblastine (VBL), and demonstrated a similar capacity to alter tubulin architecture. However, distinct from PTX, the connection with impaired ADP bioenergetics is seemingly independent of the interaction between tubulin and VDAC [118]. Rather, the mechanism may be reflective of the lesser-known role of VBL as an inhibitor of microtubule-associated proteins, 1A/1B light chain 3B (LC3B)-II, degradation. Thus, VBL is a likely suppressor of auto-lysosomal maturation and autophagic flux [124]. Further investigation of taxanes and vinca alkaloids with regard to skeletal muscle health is warranted, given the association with microtubule perturbations and induction of dystrophic phenotypes [125].

## 3. Therapeutic Strategies to Mitigate Chemotherapy-Induced Cachectic s Myopathy: An Update

**Figure 4:**
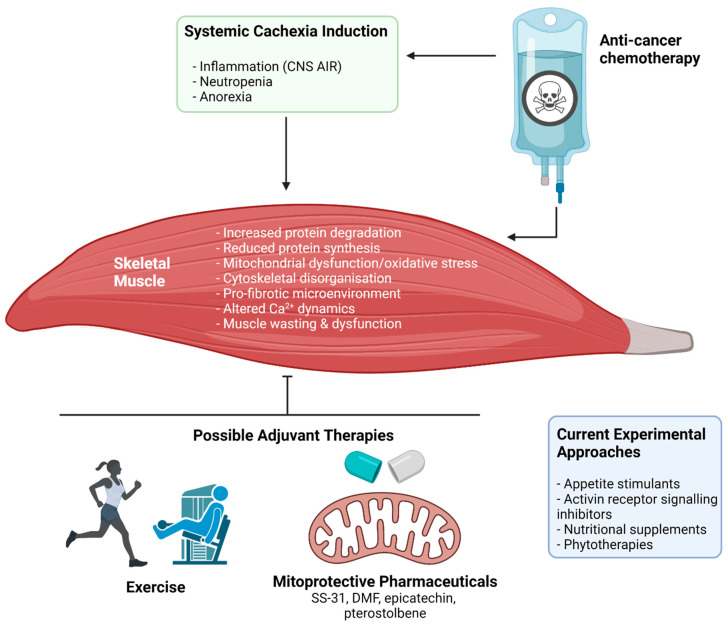
The impact of anti-cancer chemotherapy treatment on cachectic myopathy and possible protective therapeutic interventions. Broadly, chemotherapeutic agents used in clinical cancer treatment can both directly and indirectly target skeletal muscle through induction or amplification of systemic cachexia. The result is the initiation of a wasting and dysfunction program within skeletal muscle, involving: increased muscle protein degradation, reduced protein synthesis, mitochondrial dysfunction and oxidative stress, cytoskeletal disorganisation and reduction of key cytoskeletal proteins that stabilise the muscle membrane, pro-fibrotic signalling within the extracellular matrix, and altered calcium (Ca2+) dynamics. The result is muscle wasting and dysfunction that leaves patients weak and fatigued, which affects their capacity to undertake activities of daily living and reduces quality of life. Several potential therapeutic approaches are currently being investigated to protect against or treat these symptoms including appetite stimulants, activin receptor signalling inhibitors, nutritional supplements, and phytotherapies. Novel therapeutic strategies could include exercise and mitoprotective compounds (e.g., SS-31, BGP-15, dimethyl fumarate (DMF), epicatechin, and pterostilbene). Abbreviations: CNS AIR: central nervous system acute illness response. Created with biorender.com (accessed on 6 July 2021).

Currently, cachexia represents a significant unmet challenge in cancer, with no treatment approved for clinical use. This is likely due to the complexity of the syndrome, especially at the skeletal muscle level. There are multiple contributing factors to the induction of myopathy during anti-cancer therapy. For example, muscle deconditioning due to hospitalisation-dependent factors such as prolonged periods of bed rest, reduced opportunity to undertake physical activity, and depressive mood/fatigue could contribute significantly to cachexia progression during chemotherapy administration [126]. Several investigations into therapeutic strategies to mitigate the debilitating effects of cancer cachexia are underway with multiple candidates showing promise. These include exercise and multi-target pharmaceutical/nutraceutical adjuvant interventions [127,128] (Figure 4). Herein, we summarise the current knowledge and provide insights surrounding these candidate strategies to better inform future investigations. In particular, this section will focus on therapeutic strategies that elicit protection against mitochondrial dysfunction, a key mechanistic event in the induction of cachectic myopathy [129,130]. Specifically, mitochondrial degeneration is suggested as an event preceding muscle wasting in cachexia [131], and thus is a prime therapeutic target for early intervention.

### 3.1. Exercise Interventions

Exercise is a non-pharmacological and cost-effective strategy that is currently being investigated for therapeutic purposes against cachexia. The therapeutic potential of exercise in the cancer setting is multi-faceted, but includes both modulation of systemic inflammation and regulatory control of the redox balance [130], two inherently related mechanisms that drive cachexia [129]. These mechanisms are also prominent drivers of chemotherapy-induced cachectic myopathy. Thus, exercise interventions could prevent the atrophy and loss of function associated with anti-cancer chemotherapy treatment. As such, exercise has been investigated in experimental models exploring its efficacy to mitigate the impact of chemotherapy (particularly DOX) on skeletal muscle health (summarised in Table 1). In general, exercise has shown modest protective efficacy against the loss of skeletal muscle mass and functionality from chemotherapy administration [132]. Smuder proposes that this is linked to the capacity of exercise to promote endogenous antioxidant expression, protein chaperoning via heat shock protein-70 (HSP-70) to mitigate proteolytic activity, and increased multi-drug resistant proteins [133]. It is important to note that the majority of studies listed in Table 1 employed exercise as a pre-conditioning strategy prior to the administration of DOX, demonstrating the capacity of exercise to prime skeletal muscle to resist DOX-induced stress [63,64,134,135,136,137,138]. However, the clinical compatibility of this approach is questionable since cancer treatment would need to be delayed while muscles were conditioned using exercise programs. There is also a lack of data on the efficacy of pre-treatment exercise strategies for other chemotherapeutic agents aside from DOX. Jones and Alfano highlight a paucity of clinical studies investigating the utility of exercise interventions in the pre-treatment stage [139]. Rather, exercise interventions throughout the cancer survivorship continuum have predominantly been studied during and post-chemotherapy treatment, as per the adapted Physical Exercise Across the Cancer Experience (PEACE) framework [139]. To date, only four pre-clinical studies have investigated the efficacy of exercise during chemotherapy administration. These studies investigated different chemotherapeutic agents and/or regimens as well as diverse exercise modalities [78,140,141,142]. This makes it difficult to form a consensus sufficient to facilitate clinical exercise prescription based upon pre-clinical data. For example, some studies employed a maximal treadmill running test to exhaustion [141,143]. This is a low-cost alternative to incorporating the use of metabolic studies that enable the quantification of peak/maximum oxygen consumption (VO_2_ peak/max), which can be used to identify a relative exercise intensity [144]. Other studies have incorporated metabolic analyses enabling calculation of a relative VO_2_ peak/max [144]. However, given the lack of consistency across different rodent models, a knowledge gap remains in translating exercise intensity criteria between rodents and humans [145]. Additionally, there are no known experimental studies assessing the utility of exercise in the post-treatment recovery stage.

Thus far, animal experiments investigating the utility of exercise programs to resist chemotherapy-induced cachectic myopathy have not been without their pitfalls. There are numerous confounding variables highlighted in Table 1 including the selection of rodent species, strain, age, gender, and muscle type. These are all factors that can influence the protective efficacy of exercise in the context of chemotherapy-induced myopathy [145,146,147,148,149]. Additionally, tissue harvest time, relative to final chemotherapy dose, is a crucial consideration since transient events versus adaptive responses can be confused when making static measurements at a fixed timepoint. It is also imperative for experimental models to evaluate the feasibility and efficacy of resistance training during chemotherapy administration, since in vivo load-induced hypertrophy has emerged as having translational potential (for review see [150]). Animal studies to date have predominately focused on endurance training. In particular, it would be of great interest to determine whether chemotherapy-treated skeletal muscle could recover from the myotrauma induced by resistance training interventions. Indeed, Huang et al. [151] demonstrates that DOX can impair the inflammatory response necessary for skeletal muscle repair and remodelling following damaged caused by eccentric-exercise. Thus, further investigation is warranted.

Experimental animal models used to investigate the efficacy of exercise interventions during the administration of chemotherapy often utilise a treatment regimen that involves the metronomic delivery of chemotherapeutic agents spread out over an extended duration. This approach, while clinically compatible, results in milder skeletal muscle effects compared to the pre-conditioning studies, which utilise a single near-maximum tolerable dosage (MTD) bolus injection of a clinically-relevant accumulated human dose [152]. However, metronomic delivery of chemotherapy is a more compatible representation of the clinical scenario. This is especially true for DOX, which is clinically administered through slow-intravenous infusion or as repeated fractional doses cyclically over several weeks, to regulate plasma concentration of the drug. As such, severe cytotoxicity is prevented while anti-cancer efficacy is maintained [153]. The utility of metronomic delivery regimens in experimental models of chemotherapy cachexia still require a balance between retaining a clinically compatible cumulative dose and maintaining survival of the animals, so that exercise training interventions can be implemented. Going forward, future studies should refer to current gold standard models published in both de Lima et al. [143] and Ballaro et al. [89], which evaluate the effect of exercise during the synergistic-induction of cachectic myopathy from both chemotherapy and cancer-related factors. If possible, a chemotherapy control group should be included alongside a cancer control group when models are being optimised. This would help inform decision making regarding choice of exercise modality and secondary targets outside of mass and function preservation (e.g., mitigating systemic inflammation or insulin resistance) [50,154,155]. Current models need to be improved to enrich the clinical interpretability of the findings, thus promoting wider translational capability.

Current clinical studies indicate that aerobic exercise training can reduce fatigue and anxiety/depressive moods to improve participation in physical activity, while resistance exercise training can improve lean mass and muscular strength [156,157,158]. Interestingly, there is only weak evidence that either exercise modality can preserve skeletal muscle mass or cross-sectional area (CSA) [156,159,160]. This may reflect difficulties associated with measuring muscle mass in humans [161] or the barriers associated with incorporating muscle biopsies in clinical trials such as high costs and low patient recruitment due to the invasiveness of sampling. Nevertheless, these data highlight inconsistency with data derived from chemotherapy treated, cancer-free mouse models, which demonstrate protective efficacy from exercise (see Table 1) [78,142]. Perhaps even more interesting, and consistent with the clinical data, is that exercise did not alter skeletal muscle mass or CSA in two tumour-burdened mouse models treated with chemotherapy [89,143]. Thus, tumour factors appear to be most influential in dictating whether skeletal muscle can be modulated by exercise. While mass may not necessarily be modifiable by exercise therapy, de Lima et al. importantly demonstrated that exercise training improved skeletal muscle recovery after the cessation of chemotherapy, a paradigm yet to be explored in clinical studies [143]. Considering that current exercise interventions only elicit modest protective efficacy against chemotherapy and cancer-induced cachectic myopathy at best, there is an emphasis to explore multi-modal therapeutic strategies that involve exercise prescription programs alongside pharmacological interventions for synergistic benefits. Furthermore, given that some cancer patients may have a reduced opportunity to undertake exercise training due to hospitalisation-related deconditioning, multi-targeted pharmacological and/or supplementary interventions strategies that mimic specific aspects of exercise should be identified and explored for their therapeutic efficacy.

### 3.2. Adjuvant Therapies

Complementary to exercise strategies, several adjuvant candidates have been evaluated for their protective potential to combat chemotherapy-induced cachectic myopathy. Drug candidates focus on different biochemical targets that are involved in diverse molecular and physiological aspects that characterise the cachectic myopathy phenotype [40]. These candidates arise from a range of different therapeutic classes including activin receptor signalling inhibitors [59,60,100,103,165], appetite stimulants [77,80,82,166,167,168,169,170], nutritional supplements [171,172,173,174,175,176], and phytotherapies [112,113,177,178,179,180,181,182,183,184,185]. Activin receptor signalling inhibitors have shown strong pre-clinical efficacy to mitigate cancer and chemotherapy-induced cachexia through preserving skeletal muscle mass and function [186]. However, there has been limited success in the clinical translation of this pharmacological target, with a Phase II trial conducted by Novartis Pharmaceuticals (NCT01433263) demonstrating that activin receptor antibody, BYM338 (Bimagrumab), improved lean mass and muscle volume, but contributed to a net loss of body mass [187].

Appetite stimulants, in particular ghrelin receptor agonists, have shown promise in the pre-clinical setting to normalise food intake and muscle mass/CSA of chemotherapy-treated mice compared to healthy counterparts [80,82,170]. These findings translated clinically as observed through the ROMANA 1 & 2 trials (NCT01387269 & NCT01387282), where anamorelin (a ghrelin receptor agonist) increased body and lean mass of advanced cancer patients. However, it was not approved for clinical use because it failed to improve grip strength—a primary endpoint of the trial [188].

Nutritional supplements including essential amino acids and fatty acids have shown the capacity to protect against cancer and chemotherapy-induced cachexia, highlighting translational potential in single supplement interventions [189]. However, nutritional supplements are only a complementary piece of the cachexia puzzle as their clinical utility is primarily dependent on the anabolic potential of the individual [190]. Additionally, nutrition-related guidelines in cancer are based mostly on expert consensus, rarely on clinical trial evidence, highlighting a greater need to investigate multi-combination nutritional supplement interventions at the clinical level [191]. Similarly, compounds from the phytotherapies class have typically not been clinically evaluated for their therapeutic efficacy to mitigate cachectic myopathy given their rare utility in Western medicine [192]. This aspect should be re-considered based on encouraging pre-clinical data—specifically, the reduction of atrogene transcription, which underscores skeletal muscle wasting and holds strong promise as a therapeutic target against cachexia [193]. 

An emerging therapeutic class of particular interest to our laboratory group, and others, is the utility of mitoprotective compounds to combat skeletal muscle oxidative stress. Enhanced ROS production is a common underlying mechanism associated with multiple chemotherapies [32,33] and is a key contributing factor to mitochondrial dysfunction, a tenet of cachectic myopathy [36,37]. To date, mitoprotective compounds have not been evaluated in clinical trials. However, multiple mitoprotective agents have been experimentally evaluated for their potential protective efficacy against chemotherapy-induced cachectic myopathy, which will be discussed herein.

One of the first mitoprotective agents investigated for its therapeutic efficacy alongside chemotherapy was SS-31, a cardiolipin-targeting peptide, which preserves mitochondrial cristae structure and promotes oxidative phosphorylation [194]. SS-31 was shown to attenuate DOX-induced activity of multiple proteolytic systems (e.g., UPS, apoptosis, and calpains) in various muscle types, which prevented skeletal muscle atrophy [56,68]. Further, SS-31 normalises DOX-induced ROS emission in C2C12 myotube cultures and rodent muscle, with the latter observed as an acute event [33,56]. However, Ballaro et al. demonstrated that SS-31 administration yielded a modest mitoprotection, and subsequently, limited therapeutic efficacy in a more clinically compatible model of cachectic myopathy. In this study, mice were injected with C26 colorectal adenocarcinoma cells and metronomically dosed with OXA and 5FU combination chemotherapy over five weeks [90]. These data highlight that SS-31 may be more efficacious at a preventative stage of cachexia (i.e., pre-cachexia) when mitochondria are less damaged [131]. Alternatively, SS-31 may elicit specific mitoprotection against the anthracycline DOX, which induces mitochondrial stress differently to platinum-based alkylating agents, albeit this would need to be confirmed in future studies of DOX utilising more clinically compatible models.

Another adjuvant mitoprotective agent is BGP-15, a hydroximic acid derivative nicotinic acid-amidoxime small molecule that can preserve skeletal muscle metabolic homeostasis and mitochondrial quality control processes [195,196]. In particular, BGP-15 has been touted as an inhibitor of poly-(ADP-ribose) polymerase-1 (PARP-1) and co-inducer of HSP-70 [197,198], which are mechanisms associated with improved mitochondrial content, function, and oxidative capacity [199,200]. Furthermore, BGP-15 has also shown pleiotropic capacity to elicit mitoprotection independent of these mechanisms [201], a likely explanation of its therapeutic potential in a range of myopathies [202,203,204,205]. Our laboratory group has evaluated the therapeutic utility of BGP-15 alongside multiple chemotherapies including OXA, IRI, and 5FU with mixed efficacy [88,99,104]. BGP-15 was protective against OXA-induced lean mass loss, while also normalising ROS generation and mitochondrial viability [88]. However, alongside IRI, BGP-15 paradoxically rescued (partially) body, lean, and skeletal muscle mass in addition to muscle contractile function, while exacerbating the IRI-induced muscle protein synthesis inhibition and reduced expression of the cytoskeletal proteins, dystrophin, and β-dystroglycan [104]. The recovery of muscle mass and function may have been due to BGP-15′s enhancement of ATP production and mitochondrial density. Indeed, the latter was also enhanced when BGP-15 was delivered alongside 5FU and was associated with improved mitochondrial fusion. Furthermore, BGP-15 suppressed the 5FU-induced phosphorylation of NF-κB and MAPK isoforms [99], two likely mechanisms involved in the induction of cachectic myopathy. Given the heterogeneity of our findings, further investigations are required with a focus on clinically compatible combination chemotherapy regimens before BGP-15 can be considered a viable therapeutic candidate to protect against cachectic myopathy.

Future studies investigating adjuvant mitoprotective candidates should consider agents that can synergistically target chemotherapy driven oxidative damage, alongside other signalling pathways involved in the induction of cachectic myopathy. One potential compound is the nuclear factor erythroid 2-related factor 2 (Nrf2) transcriptional activator, dimethyl fumarate (DMF). DMF is a methyl ester of fumaric acid purported to upregulate cytoprotective response genes, and suppress NF-κB signalling, which imparts an anti-oxidative and -inflammatory effect [206] Another Nrf2 activator with therapeutic potential in this setting could be pterostilbene [207], a dimethoxylated analogue of resveratrol, a compound with demonstrated efficacy against DOX-mediated skeletal and cardiac myopathy [208,209]. Like resveratrol, pterostilbene can prolong lifespan, mitigate oxidative stress and normalise dysregulated autophagy in experimental models [210,211]. However, pterostilbene has greater bioavailability and a longer half-life compared to resveratrol [212], suggesting greater translational potential. Finally, epicatechin is another potentially viable mitoprotective candidate, which activates Nrf2 to inhibit oxidative stress [213]. Interestingly, epicatechin has been shown to restore the expression of dystrophin and other key cytoskeletal proteins in models of myopathy such as Becker muscular dystrophy and diabetes [214,215]. This highlights the protective potential of epicatechin against the chemotherapy-induced reduction of dystrophin expression shown by our group [99,104], and in cancer-induced cachectic myopathy in which dystrophin is also reduced [216]. Importantly, the adjuvant candidates proposed are not associated with cancer growth, and thus are unlikely to impact the anti-cancer efficacy of chemotherapy treatment [206,217,218].

Other compounds have also been evaluated for their therapeutic efficacy against chemotherapy-induced cachectic myopathy, whereby mitoprotection is elicited as a secondary effect to the central mechanism. Sodium nitrate (SN) supplementation is one of these strategies. SN potentiates the nitrate/nitrite/nitric oxide (NO) pathway to increase endogenous NO production as the central mechanism [219]. Mitoprotection occurs concurrently via an alternate pathway [220]. SN is cardioprotective during DOX treatment in mice, with the preservation of left ventricular function dependent on the mitigation of oxidative stress and mitochondrial Complex I dysfunction [221,222]. However, our evaluation of SN supplementation alongside DOX administration failed to elicit a protective effect against cachectic myopathy [223]. Interestingly, the metabolic cytoprotectant, metformin, has also been investigated alongside DOX and demonstrated no protective benefit against chemotherapy-induced cachectic myopathy. These drug candidates both promote adenosine monophosphate-activated protein kinase (AMPK) signalling to preserve cellular energy status [220,224]. Thus, they may have greater utility as a therapeutic strategy to mitigate aberrant skeletal muscle glucose uptake during chemotherapy treatment.

## 4. Future Directions and Conclusions

Chemotherapy is an under-appreciated contributing factor in the induction of cachectic myopathy. To date, research has predominately contextualised cancer cachexia as governed by tumour-related factors without considering the other side of the cachexia sphere—chemotherapy [9]. This is problematic with respect to the clinical compatibility of this paradigm, as the large majority of cancer patients typically receive chemotherapy as part of their treatment strategy. This highlights the need for experimental studies to reflect the synergistic insult from cancer and chemotherapy in the induction of cachectic myopathy [15]. Models that allow the exploration of both factors in combination, and, using the spectra of chemotherapeutic agents, multi-therapy regimens, and cancer sub-types, are required. Future investigations in this area also need to consider the impact of novel chemotherapeutic agents in clinical trials across all cancers. For example, the therapeutic utility of multi-kinase inhibitors is becoming increasingly prevalent [225] and they demonstrably have cachexia-inducing properties [226,227]. Additionally, drug compounds that are utilised to mitigate the side-effects of clinical chemotherapy treatment such as the well-described gastrointestinal toxicities elicited by dexamethasone [228] need to be stratified for their capacity to potentiate chemotherapy-induced cachectic myopathy [229,230].

The utility of a cachexia scoring system within investigative animal research such as the animal cachexia score (ACASCO) [231] is warranted to justify a relative cachectic burden concerning chemotherapy- and cancer-induced myopathy. This will assist with the clinical interpretation of experimental findings, and where they fit within the cachexia diagnostic continuum in human patients, which incorporates three key stages: pre-cachexia, cachexia, and refractory cachexia [14]. Specific to chemotherapy-induced cachectic myopathy, a modified scoring system should also be considered to incorporate biomarkers of oxidative stress and skeletal muscle damage, which are currently being developed for other severe skeletal myopathies that share similar features with cachectic myopathy (i.e., dystrophin loss and oxidative stress) as highlighted by Grounds et al. [232].

Despite emerging as a burgeoning sub-field of skeletal muscle wasting conditions, chemotherapy-induced myopathy is, to date, a pariah with respect to research effort and directed funding in contrast to cancer-induced myopathy [9]—comparatively, it remains on the ‘dark side’ of the cachexia sphere. Illuminating the mechanisms involved and the physiological repercussions as well as actively pursuing protective therapeutics will enrich clinical decision making, patient outcomes, and the quality of cancer survivorship.

## Figures and Tables

**Table 1:** Experimental studies on the effect of exercise against chemotherapy-induced cachectic myopathy.

Study	Animal Information	Exercise Modality	Chemotherapy Model	Key Observations
Smuder et al., 2011 [63]	6-month-old male Sprague-Dawley rats	TR: F: 5 days; I: 30 m/min; D: 60 mins/day; T: 0° incline.	1 × IPI 20 mg/kg of DOX post-exercise. Harvest 24 h post-IPI.	TR normalised oxidative stress and damage, calpain activity and proteolysis of actin in SOL muscles.
Smuder et al., 2011 [64]	6-month-old male Sprague-Dawley rats	TR: F: 5 days; I: 30 m/min; D: 60 mins/day; T: 0° incline.	1 × IPI 20 mg/kg of DOX post-exercise. Harvest 24 h post-IPI.	TR normalised autophagy activity in SOL muscles.
Bredahl et al., 2016 [134]	Male Sprague-Dawley rats. Age not specified	1. TR with progressive overload: F: 5 days/week, for 10 weeks; I: 20 to 30 m/min; D: 20 to 60 min/day; T: incline 0 to 18°. 2. CHL: Progressive food and water elevation to stimulate voluntary bi-pedal standing or jumping [162].	1 × IPI 15 mg/kg of DOX post-exercise. Harvest 5 days post-IPI.	TR was not protective against body mass loss and exacerbated EDL muscle mass loss. RT did not alter body or skeletal muscle mass but prevented SOL contractile dysfunction.
Kavazis et al., 2014 [135]	6-month-old male Sprague-Dawley rats	TR: F: 5 days; I: 30 m/min; D: 60 mins/day; T: 0° incline.	1 × IPI 20 mg/kg of DOX post-exercise. Harvest 24 h post-IPI.	TR normalised MuRF-1 and myostatin expression.
Mackay et al., 2019 [136]	5-week-old male C57BL/6 mice	TR: F: 5 days; I: 70% of maximal speed; D: 60 mins/day; T: 0° incline.	1 × IPI 15 mg/kg of DOX post-exercise. Harvest 72 h post-IPI.	TR partially mitigated body mass loss. TR did not modulate DOX-induced iron dysregulation.
Morton et al., 2019 [137]	6-month-old female Sprague-Dawley rats	TR: F: 10 days; I: 30 m/min; D: 60 mins/day; T: 0° incline.	1 × IPI 20 mg/kg of DOX post-exercise. Harvest 48 h post-IPI.	TR normalised DIA CSA, oxidative stress and reduced mitochondrial accumulation of DOX.
Huertas et al., 2020 [138]	6-month-old female Sprague-Dawley rats	TR: F: 5 days/week, for 2 weeks; I: 30 m/min; D: 60 mins/day; T: 0° incline.	1 × IPI 20 mg/kg of DOX post-exercise. Harvest 48 h post-IPI.	TR normalised SOL dysfunction and the transcription of AChR isoforms, δ and γ.
Dickinson et al., 2017 [140]	8-week-old ovariectomised female Sprague–Dawley rats	TR with progressive overload: F: 5 days/week, for 7 weeks, starting 1-week pre-IPI and finishing 5 days post-IPI; I: 20 to 25 m/min; D: 30–40 min/day; T: 0 to 10° incline.	1 × IPI every 2 weeks, for 4 weeks of 4 mg/kg DOX. Harvested 5 days post-IPI.	Normalised REDD1 expression. No effect on body mass or SOL muscle mass.
de Lima et al., 2018 [141]	8–10-week-old male C57BL/6 mice	TR: F: 5 days/week, for 6 weeks; I: 60% of maximal speed D: 60 mins/day; T: 0° incline.	2 × IPI per week for 6 weeks of 2.5 mg/kg DOX. Harvest not described relative to final IPI.	TR did not mitigate glucose intolerance, reduced body or GSN mass or protein synthesis. However, TR normalised corticosterone levels, autophagy activity and ambulatory function.
Hojman et al., 2014 [142]	8–12-week-old female NMRI mice	VWR: VEH: 60–80 km/mouse/week; CDDP: 10–50 km/mouse/week.	1 × IPI per week of 4 mg/kg CDDP for 6 weeks. Harvest 7 days post final-IPI.	VWR partially mitigated the loss of lean and TA mass and reduced atrogene expression. Did not protect against body or fat mass loss.
Sakai et al., 2017 [78]	8–9-week-old male C57BL/6 mice	TR: F: once a day for 9 days–5 days/week pre-CDDP and 4 days/week during CDDP week; I: 15 m/min; D: 20 min/day; T: 0° incline.	1 × IPI 3 mg/kg CDDP daily for 4 days. Harvest 24 h post-IPI.	TR did not alter body mass loss, but partially normalised QD mass and CSA, and atrogene expression.
de Lima et al., 2020 [143]	8–10-week-old male C57BL/6 mice with LLC [163]	TR: F: 5 days/week, for 2–3 weeks; I: 60% of maximal speed; D: 60 mins/day; T: 0° incline.	2 × IPI per week for 6 weeks of 2.5 mg/kg DOX. Harvest 24 h or 1-week post-IPI.	TR did not alter body mass, but enhanced GSN re-growth, normalised inflammation and atrogene expression, and enhanced tumour volume reduction.
Ballaro et al., 2019 [89]	6-week-old female Balb/c mice with c26 [164]	MWR: F: once a day, with 3 days on followed by 1 day of rest for 4 weeks; I: 11 m/min; D: 45 mins/day; T: 0° incline.	1 × IPI per week of OXA 6 mg/kg and 5FU 50 mg/kg, for 3 weeks starting at day 7 of tumour implantation. Harvest 7 days post final IPI.	MWR did not alter GSN mass, but normalised atrogene expression and mitochondrial perturbations.

Abbreviations: 5FU: 5-fluorouracil; AChR; acetylcholine receptor; c26: c26 adenocarcinoma model; CDDP: cis-diamminedichloroplatinum(II) (cisplatin); CHL: chronic hind-limb loading; CSA: cross-sectional area; DIA: diaphragm; DOX: doxorubicin; D: duration; EDL *extensor digitorum longus*; F: frequency; GSN: *gastrocnemius*; I: intensity; IPI: intraperitoneal injection; LLC: Lewis-lung carcinoma model; MWR: motorised wheel running; OXA: oxaliplatin; QD: quadriceps; REDD1: regulated in development and DNA damage response 1; SOL: *soleus*; T: type; TA: *tibialis anterior*; TR: treadmill running; VWR: voluntary wheel running.

## Data Availability

Not applicable.
